# Construction and Immunogenicity of Modified mRNA-Vaccine Variants Encoding Influenza Virus Antigens

**DOI:** 10.3390/vaccines9050452

**Published:** 2021-05-03

**Authors:** Ekaterina V. Starostina, Sergei V. Sharabrin, Denis N. Antropov, Grigory A. Stepanov, Georgiy Yu. Shevelev, Anna E. Lemza, Andrey P. Rudometov, Mariya B. Borgoyakova, Nadezhda B. Rudometova, Vasiliy Yu. Marchenko, Natalia V. Danilchenko, Anton N. Chikaev, Sergei I. Bazhan, Alexander A. Ilyichev, Larisa I. Karpenko

**Affiliations:** 1 State Research Center of Virology and Biotechnology “Vector”, Koltsovo, 630559 Novosibirsk, Russia; sharabrin.sv@gmail.com (S.V.S.); rudometov_ap@vector.nsc.ru (A.P.R.); borgoyakova_mb@vector.nsc.ru (M.B.B.); andreeva_nb@vector.nsc.ru (N.B.R.); marchenko_vyu@vector.nsc.ru (V.Y.M.); danilchenko_nv@vector.nsc.ru (N.V.D.); bazhan@vector.nsc.ru (S.I.B.); ilyichev@vector.nsc.ru (A.A.I.); lkarpenko@ngs.ru (L.I.K.); 2 Institute of Chemical Biology and Fundamental Medicine, Siberian Branch of the Russian Academy of Sciences, 630090 Novosibirsk, Russia; antr0povdn@yandex.ru (D.N.A.); stepanovga@niboch.nsc.ru (G.A.S.); metatezis@gmail.com (G.Y.S.); lemza.ae@yandex.ru (A.E.L.); 3 Institute of Molecular and Cellular Biology, Siberian Branch of the Russian Academy of Sciences, 630090 Novosibirsk, Russia; chikaev@mcb.nsc.ru

**Keywords:** mRNA-vaccine, influenza virus, mRNA modification, Anti-Reverse Cap Analog, pseudouridine, N6-methyladenosine, 5-methylcytosine

## Abstract

Nucleic acid-based influenza vaccines are a promising platform that have recently and rapidly developed. We previously demonstrated the immunogenicity of DNA vaccines encoding artificial immunogens AgH1, AgH3, and AgM2, which contained conserved fragments of the hemagglutinin stem of two subtypes of influenza A—H1N1 and H3N2—and conserved protein M2. Thus, the aim of this study was to design and characterize modified mRNA obtained using the above plasmid DNA vaccines as a template. To select the most promising protocol for creating highly immunogenic mRNA vaccines, we performed a comparative analysis of mRNA modifications aimed at increasing its translational activity and decreasing toxicity. We used mRNA encoding a green fluorescent protein (GFP) as a model. Eight mRNA-GFP variants with different modifications (M0–M7) were obtained using the classic cap(1), its chemical analog ARCA (anti-reverse cap analog), pseudouridine (Ψ), N6-methyladenosine (m6A), and 5-methylcytosine (m5C) in different ratios. Modifications M2, M6, and M7, which provided the most intensive fluorescence of transfected HEK293FT cells were used for template synthesis when mRNA encoded influenza immunogens AgH1, AgH3, and AgM2. Virus specific antibodies were registered in groups of animals immunized with a mix of mRNAs encoding AgH1, AgH3, and AgM2, which contained either ARCA (with inclusions of 100% Ψ and 20% m6A (M6)) or a classic cap(1) (with 100% substitution of U with Ψ (M7)). M6 modification was the least toxic when compared with other mRNA variants. M6 and M7 RNA modifications can therefore be considered as promising protocols for designing mRNA vaccines.

## 1. Introduction

It is important to construct vaccines against influenza viruses. To protect the population against influenza, healthcare systems use attenuated (live) or inactivated vaccines (approved by the World Health Organization) after genetically analyzing circulating seasonal influenza A and B virus strains. However, due to the high variability of influenza viruses, such vaccines are inefficient against drifting seasonal and pandemic viruses. Therefore, influenza virus composition should be changed annually, yet the process of producing an influenza vaccine is time-consuming. Consequently, a number of research teams and pharma companies have carried out studies aimed at developing a universal vaccine against the influenza virus. There are different approaches to constructing a universal influenza vaccine, e.g., developing recombinant protein immunogens, using conserved influenza virus antigens (hemagglutinin stem, and proteins NP, PB1, M1, and M2), or developing new types of vaccines [1,2,3,4,5,6].

One of the most promising approaches for constructing vaccines against highly variable viruses is to base vaccines on nucleic acids. The possibility of using mRNA and plasmid DNA for inducing cell and humoral immune response was demonstrated in the early 1990s [7,8]. The application of vaccines based on nucleic acids is promising for the prevention of infectious diseases; hundreds of clinical trials have proven this. However, until 2020, no such vaccines were approved for use in humans. The main issues when applying DNA vaccines in clinical trials includes the low transfection of human cells in vivo, weak immunogenicity, and the necessity for repeated booster vaccinations with high doses of DNA, as well as the theoretical possibility of integration into the genome. The main problems of the use of RNA include an unstable molecule and ineffective delivery, which have been partially solved in recent years. This method, however, is justified by the successful results of several developers constructing mRNA-vaccines against COVID-19. Currently, Moderna finished Phase I clinical trials of an mRNA-vaccine against seasonal influenza [9,10,11,12].

Previously, we demonstrated one possible approach for developing a universal influenza vaccine based on constructing artificial antigens containing conserved fragments of hemagglutinin and conserved protein M2 [13]. Eukaryotic plasmid vectors were used to obtain DNA vaccines encoding genes of relevant immunogens. We showed that these DNA vaccine constructs induced responses for specific antibodies and cytotoxic T-lymphocytes, providing cross-protection of mice against fatal infection with two influenza virus strains: A/California 4/09 (H1N1pdm09) and A/Aichi/2/68 (H3N2) [13].

Among the currently developed vaccines, those based on mRNA are of special interest. These vaccines have an advantage over others, including DNA vaccines. They are non-infectious, can activate both cell and humoral immune responses, and can be rapidly produced [14]. In contrast to DNA vaccines, there is no potential for integration into the cell genome in the case of RNA vaccines [15,16,17]. The mRNA vaccine technique has been actively developed over the last 3–4 years. According to an expert review of American and European researchers, an mRNA vaccine against a topical influenza strain in the case of an epidemic can be developed and produced in 1 month to immunize a country as large as the USA. In 2020, American developers of the mRNA vaccine against SARS-CoV-2 (Moderna Inc., Norwood, MA, USA together with NIAID) constructed the vaccine prototype mRNA-1273 in an unprecedentedly short amount of time. It took only 63 days from the selection of the virus sequence to construct the vaccine and conduct a Phase I clinical trial with inoculation of three doses to 45 volunteers over 6 weeks. This led to the initial data on vaccine safety and demonstrated a desired immune response.

Many studies [18,19] have demonstrated that mRNA-based vaccines are a promising platform with such useful features as flexibility, scalability, cheap production, and independence from the cold chain [15]. Due to the possibility of making a simple change to the target gene in an mRNA vaccine without modifying production technology, one can quickly respond to the emergence of new pandemic infectious agents, especially the influenza virus.

However, in vivo instability of mRNA vaccines is a significant disadvantage caused by the effect of cell enzymes. Modifying the RNA molecule can solve the problem of stability. Critically important modifications necessary for translating a protein from an mRNA molecule include a cap on the 5′-terminus, 5′ and 3′ UTR, and a poly(A)tail on the 3′-terminus. To enhance the stability of mRNA in vivo, one may use analogs of standard nucleotides, e.g., pseudouridine, that increase stability against the effects of RNases, masking it from toll-like receptors (TLR) by decreasing the activation of innate immunity [20].

It is known that modified nucleotides are present in eukaryotic cells. For example, pseudouridine (Ψ) is a part of tRNA, as well as mRNA and dsRNA. It is used by the cell system for the stabilization of long-lived RNAs such as tRNA and rRNA. Another type of modified nucleotides includes methylated nucleotides, e.g., 5-methylcytosine (m5C) and 6-methyladenosine (m6A). m6A methylation is the most common type of native RNA modification. mRNA methylation serves a regulatory function and plays an important role in regulating translation [21,22]. In eukaryotic cells, these modifications take place posttranscriptionally and are important for distinguishing native and foreign RNAs. Moreover, pseudouridine and m6A are markers of nuclear maturation of intracellular RNA. Several studies demonstrated that the inclusion of pseudouridine makes it possible to “mask” artificial RNAs for their further functioning in mammal cells [23]. Pseudouridine and its methylated derivation N1-methyl pseudouridine are actively used for constructing artificial mRNAs, including mRNA vaccines [10,20].

In most studies, mRNA modifications are limited by 100% substitution of uridine with pseudouridine. Therefore, it was of interest to assess the impact of other modified nucleotides (m6A and m5C) both on stability and translational activity of mRNA in vitro, as well as mRNA immunogenicity in vivo.

**Figure 1:**
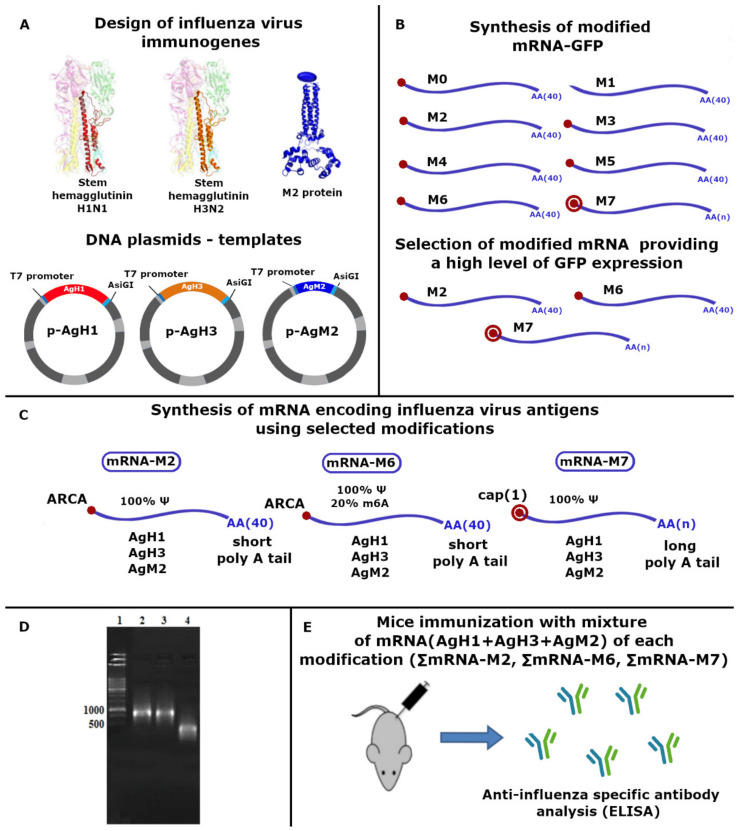
Experimental scheme. (**A**) DNA-template constructs encoding artificial antigens AgH1, AgH3, and AgM2 designed on the basis of conservative hemagglutinin stem fragments of two influenza A virus subtypes, H1N1 and H3N2, as ell as conservative M2 protein. (**B**) Synthesis and selection of modified mRNA-GFP: M0—A, G(+ARCA), C, U; M1—A, G, C, Ψ 100%; M2—A, G(+ARCA), C, Ψ 100%; M3—A, G(+ARCA), C, Ψ 50%; M4—A, G(+ARCA), C, Ψ 50%, m5C 50%; M5—A, G(+ARCA), C, Ψ 50%, m5C 50%, m6A 20%; M6—A, G (+ARCA), C, Ψ 100%, m6A 20%; M7—A, G, C, Ψ 100% + Cap(1) and Poly(A). (**C**) Synthesis of mRNA encoding influenza virus antigens using selected modifications. (**D**) Electrophoresis in 2% agarose gel. Control of matrix RNA synthesis from the corresponding DNA matrix. 1—marker M12, 2—RNA (AgH1), 3—RNA (AgH3), 4—RNA (AgM2) C. (**E**) Mice immunization with a mixture of mRNA (AgH1 + AgH3 + AgM2) of each modification (mRNA-M2, mRNA-M6, and mRNA-M7).

In this study, we conducted a comparative analysis of the biological activity of mRNA modifications encoding artificial immunogens that contained conserved fragments of the hemagglutinin stem for two influenza virus subtypes: H1N1 and H3N2, as well as conserved protein M2. We compared the effect of using the chemical cap analog ARCA (anti-reverse cap analog) and the enzyme system of capping. We also examined the effect of pseudouridine and N6-methyladenosine on the ability to provide effective protein synthesis in vitro, as well as the ability to provide an immune response. The experimental scheme is depicted in Figure 1.

The impact of modified nucleotides on RNA stability and protein translation efficiency was assessed in the model showing mRNA encode GFP. Immunogenicity of the selected modifications was carried out in the model of mRNA-vaccines encoding influenza virus antigens and by the detection of virus-specific antibodies in ELISA. We believed that this study can be useful for improving mRNA vaccine techniques.

## 2. Materials and Methods

### 2.1. Design of Immunogens and Acquisition of Plasmids Encoding Influenza Virus Antigens Used for mRNA Synthesis

To synthesize mRNA vaccine constructs, we used a template from DNA plasmids encoding the previously designed antigens AgH1, AgH3, and AgM2, which were constructed based on conserved fragments of the hemagglutinin stem of two influenza virus subtypes: H1N1 and H3N2, as well as conservative virus protein M2 [13]. Briefly, the design of the AgH1 (H1N1) antigen structure was carried out based on the hemagglutinin of influenza A virus A/Puerto Rico/8/1934(H1N1) [24], according to the algorithm described by Squires et al. [25] with modifications described by Bazhan et al. [13]. A common structure of the constructed immunogen (a) and relevant amino acid sequence (b) appears as follows:

(a) leader peptide–HA1_18-41_–gsa–HA1_290-323_–gsagsa–HA2_541-613_–transmembrane and cytosolic fragments
(b) **MKANLLVLLCALAAADA**—DTVDTVLEKNVTVTHSVNLLEDSHgsaNSSLPYQNTHPTTNGESPKYVRSAKLRMVTGLRNgsagsaTQNAINGITNKVNTVIEKMNIQDTATGKEFNKDEKRMENLNKKVDDGFLDIWTYNAELLVLLENERTLDAHDS—NVKNLYEKVKSQLKNNAKEIGNGCFEFYHKCDNECMESVRNGTYDYPKYSEESKLNREKVDGVKLESMGIYQILAIYSTVASSLVLLVSLGAISFWMCSNGSLQCRICI

Here, **MKANLLVLLCALAAADA** is a leader peptide. Lowercase letters denote amino acid residues corresponding to linker peptides. HA118–41, HA1290–323, and HA2541–613 are fragments forming around the hemagglutinin stem. The C-terminal transmembrane and cytosolic fragments are separated by a hyphen.

The artificial antigen AgH3 (H3N2) was developed based on the structure of the AgH1 antigen. However, in this case, its amino acid sequence was designed as a consensus sequence on the basis of aligning the sequences of hemagglutinin from several H3N2 strains [13]:

**MKTIIALSYILCLVFAQ**—TIVKTITNDQIEVTNATELVQSSSgsaPNDKPFQNVNRITYGASPRYVKQNTLKLATGMRNgsagsaTQAAINQINGKLNRLIGKTNEKDHQIEKEFSEDEGRIQDLEKYVEDTKIDLWSYNAELLVALENQHTIDLTDS—EMNKLFERTKKQLRENAEDMGNGCFKIYHKCDNACIGSIRNGTYDHDVYRDEALNNRFQIKGVELKSGYKDWILWISFAISCFLLCVALLGFIMWACQKGNIRCNICI.

The antigen AgM2 was presented by a conserved sequence of the M2 protein of virus A/Wisconsin/67/2005(H3N2) (EU100611.1).

Genes encoding the designed antigens were cloned into the vector plasmid pcDNA3.1. As a result, we obtained three recombinant plasmids: p-AgH1, p-AgH3, and p-AgM2 (Figure 1A).

Plasmid peGFP-N1 (https://www.addgene.org/vector-database/2491 (accessed on 30 April 2021)), with a gene encoding green fluorescent protein (GFP), was used as the template for mRNA-GFP synthesis to control the efficiency of different modifications.

### 2.2. Synthesis and Purification of eGFP mRNAs with Different Nucleotide Modifications

Plasmid peGFP-N1 was used as the template for eGFP synthesis in modified RNAs. Primers used in the work are listed in Table 1, wherein the reverse primer encoded a 40-nucleotide poly(A)tail. PCR products were amplified after the following protocol: 95 °C, 5 min; 95 °C, 10 s; 58 °C, 10 s; and 72 °C, 20 s for 30 cycles. The templates were purified using a MinElute PCR Purification Kit (Qiagen, Germantown, MD, USA). The mRNAs were synthesized using a highly effective in vitro RNA synthesis kit (Biolabmix, Novosibirsk, Russia) with NTP mixes containing different NTP modifications M0–M6 (see Table 2), such as pseudouridine (Ψ), 5-methylcytosine (m5C), or N6-methyladenosine(m6A) (BioSan, Novosibirsk Russia), as well as ARCA (TriLink BioTechnologies, San Diego, CA, USA). Moreover, 450 ng of amplicons per reaction were used for mRNA synthesis. Each modified transcript was treated with 2 U per 100 μL of the reaction of DNase I (ThermoFischer Scientific, Waltham, MA, USA) and the same amount of FastAP Thermosensitive Alkaline Phosphatase (ThermoFischer Scientific, Waltham, MA, USA). It was then purified using a kit for RNA extraction via bacterial, mammalian, and epithelial cells (Biolabmix, Novosibirsk, Russia). For M7 modification synthesis, we used the linearized DNA template for peGFP-N1 on restriction site XmaI. Polyadenylation and capping were carried out using a ScriptCap™ Cap 1 Capping System (CellScript, Madison, WI, USA) and an A-Plus™ Poly(A) Polymerase Tailing Kit (CellScript, Madison, WI, USA) according to the manufacturer’s recommendations. mRNA purification was carried out using a Monarch^®^ Total RNA Miniprep Kit (New England Biolabs, Ipswich, MA, USA).

### 2.3. HEK293FT eGFP mRNA Transfection

HEK293FT cells grew up to 40–50% confluence in a DMEM/F12 medium. On transfection day, 2 μL of Lipofectamine 3000 (ThermoFischer Scientific, Waltham, MA, USA) was mixed with 1.2 μg of each modified mRNA and incubated for 15 min to induce the formation of the Lipofectamine: mRNA complex.

Then, the DMEM medium without serum (500 μL) was mixed with lipoplexes. The cell-growth medium was decanted and changed with the lipoplex-containing medium. The cell plates were stored in a CO_2_ incubator for 3 h at 37 °C. Afterward, the medium containing 20% serum was added to the serum-deficient medium at a ratio of 1:1. The cell plates were then stored in a CO_2_ incubator for 24 h prior to microscopy analysis. Results were visualized using an Olympus CKX53 microscope or flow cytometry. To detect the level of GFP expression, 20,000 events per sample were processed in separate cells and obtained using a Ze5 flow cytometer (Bio-Rad Laboratories Inc., Hercules, CA, USA). Finally, they were analyzed in FlowJo software.

### 2.4. Synthesis of mRNAs Encoding Influenza Virus Antigens

mRNA synthesis that encoded AgH1, AgH3, and AgM2 antigens was conducted using relevant DNA templates in three modifications: mRNA-(X)-M2, mRNA-(X)-M6, and mRNA-(X)-M7, where X denotes each of AgH1, AgH3, and AgM2 constructs. The synthesis protocol is described above, depending on the modification. In the case of M2 and M6 modifications, we used DNA amplicons as a template (primers for their acquisition are indicated in Table 1). In the case of M7, we used plasmids linearized on the AsiGI restriction site.

### 2.5. Immunization of Animals

Work with animals was carried out according to the “Guide for the Care and Use of Laboratory Animals”. The protocols were approved by the Institutional Animal Care and Use Committee (IACUC) affiliated with the State Research Center of Virology and Biotechnology “Vector” (Permit Number: SRC VB “Vector”/10-09.2020).

To evaluate mRNA-vaccine immunogenicity, we used BALB/c female mice who weighed 16–18 g. Mice were divided into five groups with 6 animals in each group. In group 1, ΣmRNA-M2, mice were immunized with a mix of mRNA(AgH1)-M2, mRNA(AgH3)-M2, and mRNA(AgM2)-M2 (45 μg RNA/100 μL of normal saline). In group 2, ΣmRNA-M6, mice were immunized with a mix of mRNA(AgH1)-M6, mRNA(AgH3)-M6, and mRNA(AgM2)-M6 (45 μg RNA/100 μL normal saline). In group 3, ΣmRNA-M7, mice were immunized with a mix of mRNA(AgH1)-M7, mRNA(AgH3)-M7, and mRNA(AgM2)-M7 (45 μg RNA/100 μL normal saline). In group 4 (positive control), mice were immunized with influenza virus A/California /07/09 (H1N1) (100 μL). In group 5 (negative control), mice were immunized with normal saline (100 μL).

Mice were immunized intramuscularly in the upper thigh of the hind limb two times on days 0 and 21. On the 41st day, mice blood was sampled for sera analysis. Sera were centrifuged (10,000 rotations per minute for 15 min) to separate it from cell elements and heated for inactivation in the complement system (for 30 min at 56 °C).

### 2.6. ELISA

We used ULTRIX with a purified influenza virus, as well as H1N1 and H3N2 antigens, to prepare for the vaccines. Antigens (1 μg/mL) were adsorbed in 96-well plates in PBS (Greiner Bio One GmbH, Frickenhausen, Germany) at 4 °C for 12 h. Then, they were washed in PBST and blocked by 1% casein solution in a wash buffer for 60 min at room temperature. After that, serum samples were added in a three-fold serial dilution starting at 1:30 and incubated for 60 min at 37 °C. After washing, we added rabbit antibodies against mice IgG conjugated with horseradish peroxidase (Sigma-Aldrich, St. Louis, MO, USA) and incubated for 60 min at 37 °C. Then, we washed the plate and added a diluted TMB substrate (Amresco LLC, Solon, OH, USA). After terminating the reaction via a stop solution, we measured the optical density at a wavelength of 450 nm using a reader device for ELISA (ChroMate Awareness Technology Inc., Palm City, FL, USA). Graphs were constructed using GraphPad Prism 6.0 and Excel 2016.

### 2.7. Cell Viability Assay (MTT)

The cytotoxicity of mRNA modifications was assessed using the MTT test [26]. HEK293FT, PC3, and A549 cells (1 × 105 cells/mL) in 100 μL cell culture were seeded in 96-well plates and incubated by night. The next day, mRNA-M0, mRNA-M2, mRNA-M6, and mRNA-M7 were added to the cells at a concentration in the range from 50 μg/mL to 0.4 μg/mL and incubated for 72 h. After 72 h of cell incubation with the tested complexes, 20 μL of the MTT solution (5 mg/mL) was added to each well and incubated for 2 h in a CO_2_-incubator. After incubation, we removed the medium and added dimethyl sulfoxide (50 μL/well) to terminate the reaction. Optical density was detected via a microplate reader Varioskan LUX (Thermo Fisher Scientific, Waltham, MA, USA) at a wavelength of 570 nm. Cell viability in the presence of complexes was calculated as follows: [(optical density of texted wells/optical density of control wells) × 100%].

## 3. Results

### 3.1. Selection of Nucleotide Modifications in mRNA eGFP Model

We initially carried out a comparative analysis of different variants of mRNA modification to detect a combination of modified nucleotides that provided efficient translation in human cells (Figure 1B). Based on the data found in the literature, we selected nucleotide modifications that provided an enhanced translation efficiency and a decrease of cytotoxicity of exogenous mRNAs during mammal cell transfection [23,27,28].

The use of pseudouridine is a basic modification of foreign mRNA that is necessary for effective protein translation [20,29]. The use of methylated adenosine and methylated cytosine also provides stabilization and additional regulation to mRNA translations [30,31,32].

We synthesized mRNA-GFP variants M0–M6, which were comprised of an analog of the cap (ARCA), m5C, m6A, and Ψ in different ratios, as presented in Table 2. The control mRNA variant with ARCA without modification of nucleotides (M0); variant with 100% substitution of U with Ψ without cap (M1); co-transcriptionally capped mRNA (ARCA mRNA) with 100% substitution of U with Ψ (M2); ARCA mRNA with 50% statistical inclusion of Ψ instead of U (M3); ARCA mRNA with 50% inclusion of Ψ and m5C (M4); ARCA mRNA with 50% inclusion of Ψ, m5C, and 20% substitution of A with m6A (M5); and ARCA mRNA with 100% inclusion of Ψ and 20% inclusion m6A (M6).

**Figure 2:**
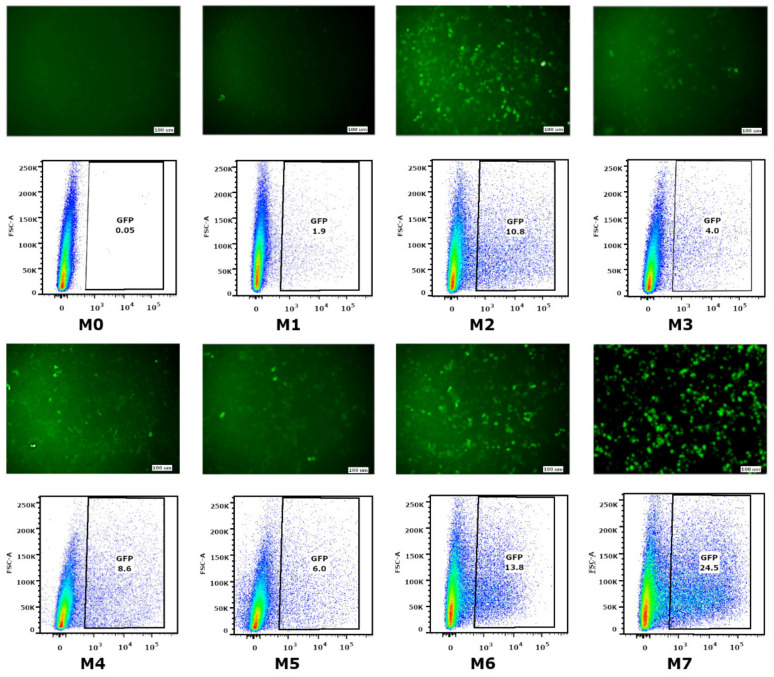
Transfection of HEK293FT cell culture with various mRNA modifications. Results were visualized 24 h after transfection with an Olympus CKX53 microscope and GFP expression in transfected cells was estimated with flow cytometry. M0—A, G(+ARCA), C, U; M1—A, G, C, Ψ 100%; M2—A, G(+ARCA), C, Ψ 100%; M3—A, G(+ARCA), C, Ψ 50%; M4—A, G(+ARCA), C, Ψ 50%, m5C 50%; M5—A, G(+ARCA), C, Ψ 50%, m5C 50%, m6A 20%; M6—A, G (+ARCA), C, Ψ 100%, m6A 20%; M7—A, G, C, Ψ 100% + Cap(1) and Poly(A).

Transfection efficiency of HEK293FT cells, obtained via mRNA, was assessed through eGFP fluorescence signal intensity, which was registered using microscopy and flow cytometry (Figure 2). The data obtained enabled us to conclude that the most efficient protein synthesis was observed in human cells when there was capping and 100% substitution of U with Ψ. The inclusion of m5C and m6A (up to 50% and 20%, respectively) failed to significantly influence mRNA translation effectiveness. Based on these results, we selected the two most efficient modification variants: M2—mRNA with 100% substitution of U with Ψ with ARCA, and M6, with the additional inclusion of 20% m6A. When co-transcriptionally comparing the capped mRNA (using ARCA) with the mRNA obtained by stages of enzymatic capping and polyadenylation (variant M7), we revealed that enzymatic modification made it possible to obtain mRNA that provided more efficient translation of eGFP protein in cells (as is seen in Figure 2). We believe that such an effect is caused by a significant difference in poly(A)tail length since the presented mRNAs were obtained from DNA templates comprised of a poly(A)tail of 40 base pairs in length without any additional enzymatic extension.

The mRNA modifications M2, M6, and M7 were selected as the most effective ones to compare against the luminescence of eGFP-transfected cells in the green spectrum. These modifications were then used to synthesize the modified AgH1, AgM2, and AgH3 mRNAs.

### 3.2. Cytotoxicity of the mRNA Modifications

**Figure 3:**
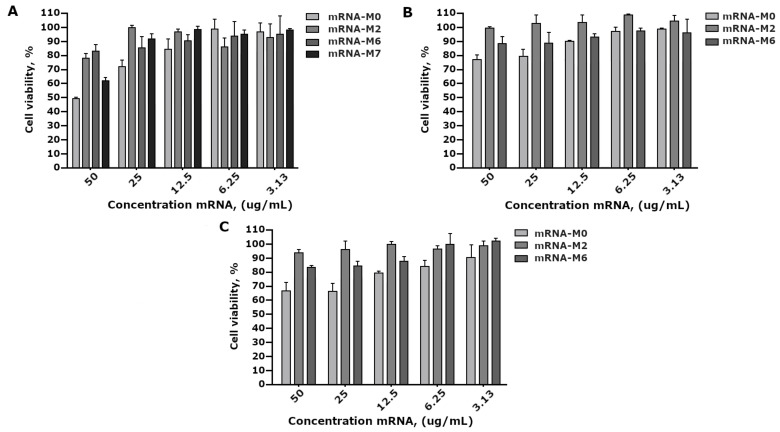
Estimation of cell viability treated with mRNA-M0, mRNA-M2, mRNA-M6, and mRNA-M7 via the MTT assay. The results are expressed as average means ± SD of triplicate experiments. (**A**) HEK293FT cells, (**B**) PC3 cells, and (**C**) A549 cells.

We investigated the effects of mRNA modifications on the viability of HEK293FT, PC3 (prostate adenoma), and A549 (lung cancer) cells using the MTT assay. The MTT test showed that for HEK293FT cell incubation with mRNA-M2, mRNA-M6, and mRNA-M7 in the concentration range from 50 to 0.4 μg/mL, the modified mRNA variants did not have a toxic effect on the cells. The proportion of viable cells at a maximum concentration of 50 μg/mL was 75, 80, and 84%, respectively. At the same time, when HEK293FT cells were incubated with mRNA-M0, at a concentration of 50 μg/mL, the proportion of viable cells was 55% (Figure 3A).

Cytotoxicity analysis in cell lines PC3 and A549 revealed results similar to those in HEK293FT cells (Figure 3B,C). We observed a decrease in the level of cytotoxic action of RNA on cells due to inclusion of modified monomers complies with our previous data, as described in [23].

### 3.3. Synthesis of mRNA Encoding Influenza Virus Antigens

We selected three modification variants aimed at enhancing mRNA translational activity (Figure 1C).

(1) mRNA-M2—100% substitution of U to Ψ, capping with ARCA
(2) mRNA-M6—100% substitution of U to Ψ and 20% addition of m6A, capping with ARCA
(3) mRNA-M7—100% substitution of U to Ψ, enzymatic polyadenylation, and capping (CellScript, Madison, WI, USA)

mRNAs that encoded influenza virus antigens were obtained as described in the Materials and Methods section, and depended on mRNA modification from three DNA templates (p-AgH1, p-AgH3, and p-AgM2). RNA synthesis control was performed by electrophoresis in 2% agarose gel (Figure 1D). An RNA of the expected size was obtained (about 850 nucleotides for AgH1 and AgH3 and 330 nt for AgM2). RNA were pooled before immunization in equal concentrations (15 μg of each mRNA per dose). We obtained the immunogen combinations of ΣmRNA-M2, ΣmRNA-M6, and ΣmRNA-M7.

### 3.4. Comparative Analysis of Immunogenicity

The combinations ΣmRNA-M2, ΣmRNA-M6, and ΣmRNA-M7 (15 μg of each immunogen per mouse) were used to immunize BALB/c mice. To assess the immunogenicity of vaccine constructs, mice were immunized twice on days 0 and 21. Five weeks after the beginning of immunization, we sampled the blood serum of mice to detect antibody titers in ELISA, specifically recognizing influenza virus antigens. Serum from mice immunized with influenza virus strains A/California/07/09 (H1N1) and normal saline were used as controls.

**Figure 4:**
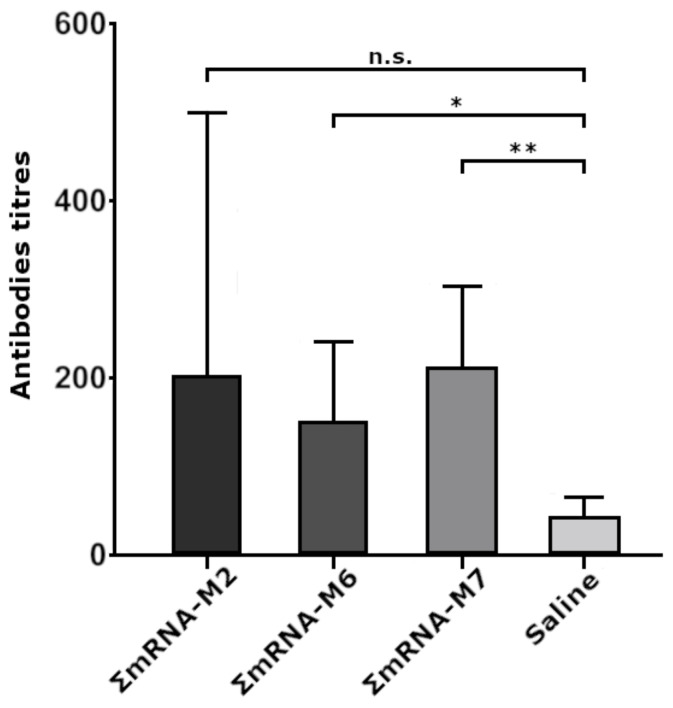
Serum antibody titers observed in BALB/c mice were immunized with a mix of modified mRNA vaccine constructs encoding influenza virus antigens. ΣmRNA-M2, ΣmRNA-M6, and ΣmRNA-M7 mice immunized with combinations of AgH1, AgH3, and AgM2 encoding mRNA with M2, M6, and M7 modifications; saline-sera mice immunized with normal saline. All animals were immunized two times on days 0 and 21. Samples of mice blood sera were collected five weeks after the first immunization. Titers of specific antibody IgG to influenza virus antigens were detected in ELISA. Statistical analysis was carried out using GraphPad Prism 6.0 software. Data are presented as mean value ± standard error of the mean. Validity was calculated using a two-way analysis of variance with multiple comparative test (n.s., statistically inaccurate; * *p* < 0.05, ** *p* < 0.01).

Data obtained using ELISA (Figure 4) showed that 2 weeks after the second immunization, titers of specific antibodies in mice immunized with the mRNA vaccine constructs increased. Statistically significant levels of antibodies were registered in the two groups immunized with ΣmRNA-M6 and ΣmRNA-M7. At the same time, we failed to detect a significant difference between mRNA modifications. In animals immunized with virus A/California/07/09 (H1N1) (positive control), antibody titers were 1:36,000.

## 4. Discussion

mRNA vaccines are a relatively new vaccine platform that has been adopted to develop therapeutic and immunoprophylactic vaccines against oncological and infectious diseases. The Moderna biotechnology company is a pioneer in designing vaccines based on mRNAs. Among the RNA vaccines constructed by Moderna, two vaccines encode full-length membrane-bound forms of the hemagglutinin of two avian influenza strains with pandemic potential: H10N8 (A/Jiangxi-Donghu/346/2013) and H7N9 (A/Anhui/1/2013). In Phase I clinical trials, both vaccines were well tolerated and induced stable humoral immune responses [10].

The use of mRNA vaccine technology makes it possible to rapidly construct vaccines against actual circulating influenza virus strains. In the current study for developing mRNA-based vaccine constructs, we used an alternative approach aimed at designing a universal influenza virus vaccine by encoding two variants of the influenza hemagglutinin stem (i.e., AgH1 and AgH3) and a conserved M2 protein (AgM2). Previously, we demonstrated that immunization of BALB/c mice with a combination of DNA vaccines encoding those antigens evoked both humoral and cellular responses, as well as a moderated statistically significant cross-protective effect against two heterologous viruses: A/California/4/2009 (H1N1pdm09) and A/Aichi/2/68 (H3N2) [13]. In this study, we converted those antigens from a DNA vaccine format to an mRNA vaccine format using different variants of nucleotide modifications. We then were able to assess their immunogenicity.

The use of mRNA for constructing immunoprophylactic vaccines demonstrates a number of attractive features, including construct simplicity, cheap production, low reactogenicity, intracellular synthesis of the target antigen, and antibody induction, as well as CD4^+^ and CD8^+^ T-cell responses [33,34]. In the course of RNA immunization, heterologous mRNA entering antigen-presenting cells immediately begins to produce foreign proteins. Yet the main problem with mRNA vaccines is their poor stability. Naked RNA is quickly destroyed by ribonucleases. For the stabilization of long-live RNA, the cell system uses modified nucleotides—e.g., Ψ, which is a part of tRNA and rRNA, as well as methylated nucleotides, such as m5C and m6A, which serve a regulatory function and play important role during translation regulation [21,22]. To synthesize encoded proteins, an RNA vaccine should evade cell systems that prevent the translation of foreign mRNAs [35]. Specifically, the organism has intracellular barriers such as TLR3, TLR7, TLR8, and retinoic acid-inducible gene I (RIG-I) of the innate immune system located in endosomal membranes and functioning against foreign mRNA [36]. However, activating these receptors could be evaded when introducing modified nucleotides [20].

Overall, together with capping and polyadenylation, introducing modified nucleotides makes it possible to regulate translation efficiency, increase RNA stability, and evade RNA-dependent cascades of inherent immunity aimed at recognizing native and foreign RNA molecules [20,30,31,32]. The basic requirement for the wide applicability of mRNA-based vaccines is the presence of all components necessary for their production at the GMP level. Currently, the majority of components are available, although some of them (e.g., enzymes for capping) are produced in limited quantities by several companies. Their analog, ARCA, is a significantly cheaper component with similar effectiveness to the classic cap. At the same time, the synthesis of cap analogs is thoroughly described, so ARCA can be synthesized by an independent laboratory with a competent staff.

In the current study, we carried out a comparative analysis of different mRNA modifications aimed at increasing translational activity. Initially, we obtained seven variants of mRNA-GFP (M0–M6) comprised of modifications with ARCA and different ratios of nucleotide analogs. In those mRNAs, we conducted full or partial substitution of natural nucleotides with their analogs, such as Ψ, m5C, and m6A. Furthermore, we obtained the eighth variant M7, which was comprised of 100% Ψ and a classic cap(1).

It is known that pseudouridine masks RNA from receptors of innate immunity [20,27]. However, the complete substitution of uridine with pseudouridine significantly stabilizes the secondary structure of mRNA, which may complicate the interaction with proteins and translation in human cells.

Therefore, we tried to include m5C and m6A in mRNA vaccines. However, most of their inclusion in mRNA composition may slow down translation [30,31,32]. Therefore, we used a relatively low share of their inclusion—up to 50% of m5C and 20% of m6A.

The ability of mRNA-GFP variants to provide GFP synthesis in eukaryotic cells was evaluated by measuring the fluorescence intensity of transfected HEK293FT cells. The low level of fluorescence was detected in cells transfected with mRNA with modifications M0, M1, M3, M4, and M5. The fluorescence of separate cells (lower than 1%) was fixed for the M0 modification (nucleotide modifications were absent) and M1 (the cap was absent). As for the M3, M4, and M5 modifications, providing partial substitution of nucleotides with Ψ, m5C, and m6A, fluorescence effectiveness was 5–8%. The highest green protein fluorescence was registered in cells transfected with mRNA-GFP with M2, M6, and M7 modifications, providing 10%, 14%, and 25% fluorescence, respectively. This suggests that 100% substitution of U to Ψ (M2 and M7) and 100% substitution of U to Ψ, as well as a 20% addition of m6A (M6), protects RNA from degradation and increases translational activity.

A comparative study of mRNA-GFP variant toxicity in HEK293FT cell culture revealed that 100% pseudouridine substitution and adding N6-methyladenine (m6A) to ARCA resulted in a decrease in toxicity; however, modifications did not significantly differ between themselves, but rather significantly differed from the control non-modified mRNA-GFP (Figure 3).

In the following steps, we determined and evaluated the immunogenicity of mRNA vaccine constructs encoding artificial influenza antigens. Synthesis templates for mRNA vaccine constructs were presented by DNA plasmids encoding the previously designed antigens AgH1, AgH3, and AgM2. These were constructed on the basis of conserved fragments of the hemagglutinin stem for two influenza A virus subtypes—H1N1 and H3N2—as well as the conserved virus protein M2. Immunization of mice with a combination of those DNA constructs induced virus-specific antibodies, with antibody titers of 1:400 determined by ELISA [13].

To immunize mice, we used combinations of naked mRNA(AgH1), mRNA(AgH3), and mRNA(AgM2) with M2, M6, and M7 modifications, providing the most intensive fluorescence of transfected HEK293FT cells. In animal groups immunized with ΣmRNA-M6 and ΣmRNA-M7, we registered a significant increase in the titers of antibody to influenza virus antigens, with an average titer of 1:200. In the ΣmRNA-M2 group, the increase in titers was insignificant, although one of the mice demonstrated a high titer of 1:800. Low antibody titers were possibly caused by the low dose of the mRNA vaccine (15 μg of each mRNA per mouse) used for immunization, or by the rapid mRNA degradation due to the absence of an mRNA delivery system. However, immunization of mice with M6 and M7 constructs induced statistically significant antibody titers. The fact that differences in antibody titers in mouse groups immunized with modified mRNA variants were statistically insignificant indicates that by at least substituting the cap with its analog, ARCA, it does not decrease the immunogenicity of modified mRNAs.

Thus, we believe that together with capping and polyadenylation inclusion of pseudouridine and m6A in the mRNA structure, it is possible to use advantages of these two natural modifications to provide RNA stability and efficient expression in human cells. Our findings revealed that modified mRNA vaccine constructs are functionally active non only in vitro but also in vivo due to the induction of synthesis of specific antibodies in immunized mice.

In our study, mRNA vaccine constructs were synthesized with the use of DNA vaccine constructs encoding influenza virus antigens (AgH1, AgH3, and AgM2) as a template [5]. Previously, we demonstrated that BALB/c mice immunization with DNA vaccines encoding those antigens induced humoral and T-cell immune responses, as well as moderate statistically significant cross-protective effect (50% and 58%) against two heterologous viruses A/California/4/2009 (H1N1pdm09) and A/Aichi/2/68 (H3N2).

We believe that obtained mRNA vaccine constructs will demonstrate protection against viral challenges (such as DNA vaccine templates) when using efficient delivery methods (i.e., electroporation, cationic polymers, dendrimers, and lipid nanoparticles). This will be the subject of our further studies.

## 5. Conclusions

Our findings revealed that modified naked mRNA vaccines encoding artificial antigens and constructed with conserved fragments of the hemagglutinin stem of influenza viruses, H1N1 and H3N2, as well as the M2 protein, can induce a specific antibody response against influenza virus in mice. The immunogenicity of mRNA vaccines in the form of naked RNA molecules, despite the use of modified nucleotides, was insufficiently high. This may have been due to their degradation by RNases and weak effectiveness of delivery in antigen-presenting cells. Considering that modified mRNA-GFP M2, M6, and M7, along with Lipofectamine, provide a sufficiently high level of GFP expression in vitro, we believe that the immunogenicity of modified mRNAs encoding influenza virus antigens will be higher when using liposomes or other cationic polymers as a means of delivery. Our further studies will be aimed at solving this issue.

## Figures and Tables

**Table 1:** Primes used in the study.

Primer	Sequence
T7-eGFP-F	5′-ATGCAGCTAATACGACTCACTATAGGATCCGCTAGCGCTACC-3′
eGFP-R40T	5′-TTTTTTTTTTTTTTTTTTTTTTTTTTTTTTTTTTTTTTTTGTAACCATTATAAGCTGCAATA-3′
forward_T7_3mG	5′-ATGCAGCTAATACGACTCACTATAAAGCTGTTCTAGAGGATCC-3′
rev_40T_3mG	5′-TTTTTTTTTTTTTTTTTTTTTTTTTTTTTTTTTTTTTTTTCCGCCTCAGAAGCCATAGA-3′

**Table 2:** Nucleotide modifications used in the synthesis of modified RNA.

**Modification**	**M0**	**M1**	**M2**	**M3**
**Base composition**	A, G(+ARCA), C, U	A, G, C, Ψ 100%	A, G(+ARCA), C, Ψ 100%	A, G(+ARCA), C, Ψ 50%
**Modification**	**M4**	**M5**	**M6**	**M7**
**Base composition**	A, G(+ARCA), C, Ψ 50%, m5C 50%	A, G(+ARCA), C, Ψ 50%, m5C 50%, m6A 20%	A, G (+ARCA), C, Ψ 100%, m6A 20%	A, G, C, Ψ 100% + cap(1)

## Data Availability

No new data were created or analyzed in this study. Data sharing is not applicable to this article.
